# Comparing clinical profiles in spondyloarthritis with Crohn’s disease or ulcerative colitis: insights from the ASAS-PerSpA study

**DOI:** 10.1093/rap/rkae064

**Published:** 2024-05-22

**Authors:** Valeria Rios Rodriguez, Tugba Izci Duran, Murat Torgutalp, Clementina López-Medina, Maxime Dougados, Mitsumasa Kishimoto, Keisuke Ono, Mikhail Protopopov, Hildrun Haibel, Judith Rademacher, Denis Poddubnyy, Fabian Proft

**Affiliations:** Department of Gastroenterology, Infectiology and Rheumatology (including Nutrition Medicine), Charité – Universitätsmedizin Berlin, Corporate Member of Freie Universität Berlin and Humboldt-Universität zu Berlin, Berlin, Germany; Clinic of Rheumatology, Denizli State Hospital, Denizli, Turkey; Department of Gastroenterology, Infectiology and Rheumatology (including Nutrition Medicine), Charité – Universitätsmedizin Berlin, Corporate Member of Freie Universität Berlin and Humboldt-Universität zu Berlin, Berlin, Germany; Department of Rheumatology, Reina Sofia Hospital, IMIBIC, University of Cordoba, Cordoba, Spain; Department of Rheumatology, Université de Paris., Hôpital Cochin, Assistance Publique, Hôpitaux de Paris, Paris, France; INSERM (U1153): Clinical Epidemiology and Biostatistics, PRES Sorbonne Paris-Cité, Paris, France; Department of Nephrology and Rheumatology, Kyorin University School of Medicine, Tokyo, Japan; Department of Nephrology and Rheumatology, Kyorin University School of Medicine, Tokyo, Japan; Department of Gastroenterology, Infectiology and Rheumatology (including Nutrition Medicine), Charité – Universitätsmedizin Berlin, Corporate Member of Freie Universität Berlin and Humboldt-Universität zu Berlin, Berlin, Germany; Department of Gastroenterology, Infectiology and Rheumatology (including Nutrition Medicine), Charité – Universitätsmedizin Berlin, Corporate Member of Freie Universität Berlin and Humboldt-Universität zu Berlin, Berlin, Germany; Department of Gastroenterology, Infectiology and Rheumatology (including Nutrition Medicine), Charité – Universitätsmedizin Berlin, Corporate Member of Freie Universität Berlin and Humboldt-Universität zu Berlin, Berlin, Germany; Department of Gastroenterology, Infectiology and Rheumatology (including Nutrition Medicine), Charité – Universitätsmedizin Berlin, Corporate Member of Freie Universität Berlin and Humboldt-Universität zu Berlin, Berlin, Germany; Epidemiology Unit, German Rheumatism Research Centre, Berlin, Germany; Department of Gastroenterology, Infectiology and Rheumatology (including Nutrition Medicine), Charité – Universitätsmedizin Berlin, Corporate Member of Freie Universität Berlin and Humboldt-Universität zu Berlin, Berlin, Germany

**Keywords:** spondyloarthritis, inflammatory bowel disease, Crohn’s disease, ulcerative colitis

## Abstract

**Objectives:**

Assuming SpA manifestations may vary among patients with different inflammatory bowel disease (IBD) subtypes, we explored the clinical characteristics associated with the presence of Crohn’s disease (CD) or ulcerative colitis (UC) in patients with spondyloarthritis (SpA).

**Methods:**

We included 3152 patients of ASAS-PerSpA study diagnosed with either axial SpA or peripheral SpA, according to their treating rheumatologist. Of these, 146 (4.6%) had confirmed IBD by endoscopy and were categorized into CD or UC groups. Demographics, clinical characteristics, treatments and patient-reported outcomes were compared between the two subgroups.

**Results:**

From 146 patients included in the current analysis, 87 (59.6%) had CD [75 (86.2%) axial SpA and 12 (13.8%) peripheral SpA], and 39 (26.7%) had UC [34 (87.2%) axial SpA and 5 (12.8%) peripheral SpA]. CD and UC groups had similar age with average of 44.9 (13.5) *vs* 44.0 (13.0) years, respectively, and a slight male predominance in CD (63.2%) compared with UC (51.3%). Diagnostic delay for SpA was 7.0 (6.9) years for CD and 8.8 (8.1) years for UC. Chronic back pain was the most reported symptom present in 95.4% of CD patients and 89.7% of UC patients. Both groups had similar musculoskeletal phenotyping, with higher frequency of psoriasis (15.4%) and uveitis 28.2% in UC; and higher tendency to be HLA-B27 positive in CD (51.9% in CD *vs*.s 39.4% in UC).

**Conclusion:**

In our analysis patients with SpA and concurrent CD or UC had mainly similar musculoskeletal phenotypes. However, they differ slightly in extra-musculoskeletal manifestations and HLA-B27 prevalence.

Key messages

- Patients with SpA and concomitant CD or UC have largely similar musculoskeletal profiles.
- Differentiating CD and UC is vital to adjust treatment strategies for effective SpA management.

## Introduction

Spondyloarthritis (SpA) includes a group of chronic immune-mediated diseases primarily affecting the axial skeleton, named axial SpA, and the peripheral joints, peripheral SpA. While the main manifestation lies in the musculoskeletal system, SpA also shares significant associations with other inflammatory conditions, including inflammatory bowel diseases (IBD) such as Crohn’s disease (CD and ulcerative colitis (UC); anterior acute uveitis and psoriasis.

The co-occurrence of SpA and IBD has been extensively documented, reflecting shared pathogenic mechanisms that result in shared treatment targets, such as biologic agents—anti-tumor necrosis factor (TNF) agents, and small molecules such as JAK inhibitors. Studies have reported a higher prevalence of IBD among patients with SpA and vice versa, with rates ranging from 6% to 14% [1–3]. When subclinical microscopic gastrointestinal inflammation is considered, the prevalence of IBD in SpA raises as high as 66% [4, 5]. This strong association has led to the inclusion of IBD as a clinical feature for the classification of SpA, encompassing both axial and peripheral forms, as defined by the Assessment of SpondyloArthritis international Society (ASAS) criteria [6, 7].

While research has traditionally focused on the concomitance of IBD as a whole in SpA, it is important to recognize that inside IBD are distinct entities with their own unique clinical features. CD is characterized by patchy transmural inflammation that can affect any part of the gastrointestinal tract, while UC primarily involves continuous inflammation of the colon and rectum, with inflammation limited to the mucosal layer [8]. These differences in the location and extent of the inflammation contribute to variations in symptoms, disease complications, and treatment strategies. Consequently, variations in the manifestations of SpA may exist among patients with these different IBD subtypes. Our manuscript seeks to explore whether there are differences in SpA characteristics and outcomes within the context of a main diagnosis of CD or UC in patients with SpA.

## Methods

### Study design and patient selection

The ASAS-PerSpA is a cross-sectional multicentre observational study conducted in a total of 24 countries. Patients with a diagnosis of SpA (*n* = 4465) were included in the study. Local rheumatologists were asked to specify the diagnosis into axial SpA, peripheral SpA, psoriatic arthritis, reactive arthritis, IBD-associated SpA, juvenile SpA, or other type of SpA. Details of the study design and description of the overall study population have been previously reported elsewhere [9].

**Figure 1.**
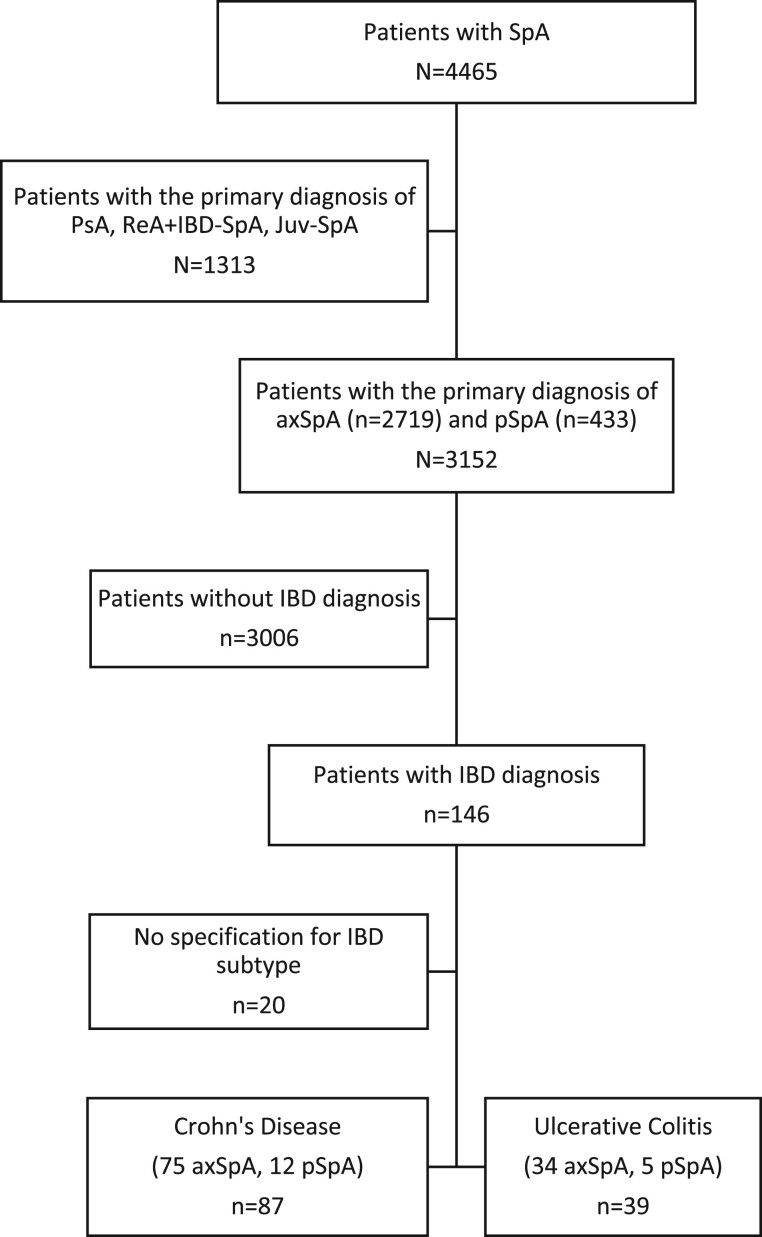
Flowchart showing the selection of patients from the PerSpA Study included in the analysis

For the present ancillary analysis, we included 3152 patients whose primary diagnosis was axial SpA or peripheral SpA, and we excluded those with the primary diagnosis of psoriatic arthritis, reactive arthritis, IBD-associated SpA, juvenile SpA, or other type of SpA (Fig. 1).

The ASAS-PerSpA was performed in accordance with the guidelines for Good Clinical Practice. Informed consent was obtained from all participants before their enrolment in the study, and the study protocol received approval from the ethical committees of all participating countries. This analysis represents an ancillary study to the primary project and, as such, did not require independent ethical approval.

### Collected variables

Data was collected by rheumatologists at each centre during a single routine patient visit using a standardized case report form. The data collected included:

- Sociodemographic information: age, sex, body mass index (BMI, kg/m^2^), smoking and alcohol consumption, and country of residence.
- Clinical characteristics: symptom duration since symptom onset, diagnostic delay. Musculoskeletal manifestations included axial and peripheral involvement. This information was detailed collected as: chronic back pain; HLA-B27 status; information on sacroiliitis based on radiographs and magnetic resonance imaging (MRI); peripheral articular disease ever; presence of objective signs of synovitis (ie, physical examination by a rheumatologist or confirmed by ultrasonography), and localization; midfoot arthritis (tarsitis) ever; ‘root-joint’ (ie, shoulder and hip) involvement ever; enthesitis ever confirmed by specific tests (ie, sonography, radiographs, MRI or bone scintigraphy); and information about dactylitis ever and localization of dactylitis (fingers or toes). Extra-musculoskeletal involvement was defined as uveitis; IBD confirmed by endoscopy and subtypes of IBD (Crohn’s disease, ulcerative colitis, unspecific); and psoriasis confirmed by a physician.
- Disease activity, functional status and patient-reported outcomes (PROs): Bath Ankylosing Spondylitis Disease Activity Index (BASDAI) [10], Bath Ankylosing Spondylitis Functional Index (BASFI) [11], Ankylosing Spondylitis Disease Activity Score-CRP (ASDAS-CRP) [12], tender joint count (TJC), 66 swollen joint count (SJC) [13], Mander enthesitis index (MEI) [14], Leeds Enthesitis Index (LEI) [15], Spondyloarthritis Research Consortium of Canada enthesitis score (SPARCC) [16], ASAS Health Index (ASAS-HI) [17], Patient Global Assessment of Well-being (PGA) (0–10), Euro quality of life (QoL)-5D (EQ-5D) [18], and the self-reported Fibromyalgia Rapid Screening Tool (FiRST) [19].
- Laboratory information: detection of C-reactive protein (CRP) levels and rheumatoid factor.
- Treatment information (current and ever): nonsteroidal anti-inflammatory drugs (NSAIDs), local and systemic corticosteroids, conventional synthetic disease-modifying antirheumatic drugs (csDMARDs) and biological DMARDs (bDMARDs).

### Statistical analysis

Our main analysis focused on comparing the clinical characteristics, disease impact and treatment modalities among patients with SpA, first divided into two groups based on the presence or absence of IBD, as confirmed by endoscopy; and then divided into groups based on the presence of CD, UC, or other forms of IBD.

Descriptive data are presented in two ways: continuous variables are presented as means with standard deviations, and categorical variables are presented as frequencies and percentages. For the univariate pairwise comparison of these variables Chi-square test was used for categorical variables, while the Mann–Whitney test and Kruskal–Wallis test were used for continuous variables. The Benjamini–Hochberg method was applied to adjust for multiple comparisons.

All data was processed and analyzed using SPSS Statistics version 25 (IBM Corp., Armonk, NY, USA). For all analyses, *p*-value less than 0.05 indicated statistical significance.

## Results

Among the 3152 patients with axial and peripheral spondyloarthritis from the ASAS Per-SpA cohort, 146 (4.6%) had IBD, as confirmed by endoscopy. The mean (SD) age of patients in the study cohort was 42.3 (13.3) years, with a higher proportion of men in the total population (65.4%) as well as in the IBD subgroup (58.2%). Body mass index (BMI) and lifestyle factors such as smoking and alcohol consumption were similar between the groups. However, the geographic distribution differed significantly, with a larger number of patients with IBD in Europe, North America, followed by the Middle East and North Africa. Asia presented significantly fewer patients with SpA and IBD than SpA without IBD (1.4% *vs*.s 24.8%). Patients with IBD had longer symptom duration and diagnostic delay for SpA compared with those without IBD [17.0 (10.1) *vs* 13.6 (11.0) years and 8.1 (7.6) *vs* 5.5 (7.6) years, respectively] as well as lower positivity of HLA-B27 (44.8% *vs* 78.1%, respectively). However, there were no significant differences between the two groups in terms of prevalence of peripheral arthritis, enthesitis or dactylitis. Patients with IBD had a higher prevalence of psoriasis and were more likely to be treated with systemic corticoids and DMARDs (conventional and biologic). Table 1 shows the sociodemographic, disease characteristics and treatment modalities of the total study population and of the subgroups with and without IBD.

**Table 1.** Socio-demographics and clinical characteristics, disease activity, and treatment of patients with SpA stratified according to the presence or absence of inflammatory bowel disease

	Total *N* = 3152	Patients with IBD *N* = 146	Patients without IBD *N* = 3006	B-H Adj. *P*
**Demographics**				
Age, years, mean (SD)	42.3 (13.3)	44.6 (13.2)	42.2 (13.2)	0.247
Sex, men, n/N (%)	2061/3152 (65.4)	85/146 (58.2)	1976/3006 (65.7)	0.350
BMI, kg/m^2^, mean (SD)	25.9 (5.2)	26.3 (4.9)	25.9 (5.2)	0.557
Ever smoker, n/N (%)	1313/3149 (41.7)	65/146 (44.5)	1248/3003 (41.6)	0.694
Ever alcohol, n/N (%)	1268/3150 (40.3)	51/146 (34.9)	1217/3004 (40.5)	0.529
Symptom duration of SpA, years, mean (SD)	13.8 (11.0)	17.0 (10.1)	13.6 (11.0)	0.023
Diagnosis delay of SpA, years, mean (SD)	5.6 (7.6)	8.1 (7.6)	5.5 (7.6)	0.023
**Extramusculoskeletal involvement**				
Psoriasis ever, diagnosed by a physician n/N (%)	238/3152 (7.6)	21/146 (14.4)	217/3006 (7.2)	0.023
Uveitis ever, n/N (%)	663/3152 (21.0)	31/146 (21.2)	632/3006 (21.0)	0.975
**Musculoskeletal involvement**				
Peripheral arthritis ever, n/N (%)	1388/3152 (44.0)	75/146 (51.4)	1313/3006 (43.7)	0.356
Enthesitis ever, n/N (%)	1361/3152 (43.2)	51/146 (34.9)	1310/3006 (43.6)	0.275
Dactylitis ever, n/N (%)	264/3152 (8.4)	6/146 (4.1)	258/3006 (8.6)	0.337
Axial involvement ever according to the rheumatologist, n/N (%)	2889/3152 (91.7)	136/146 (93.2)	2753/3006 (91.6)	0.707
Back pain, n/N (%)	3002/3152 (95.2)	141/146 (96.6)	2861/3006 (95.2)	0.678
**Laboratory assessment**				
HLA-B27 positive, n/N (%)	1906/2484 (76.7)	47/105 (44.8)	1859/2379 (78.1)	0.023
CRP mg/l, mean (SD)	12.0 (26.4)	15.1 (41.2)	11.8 (25.5)	0.578
**Treatment**				
NSAIDs, n/N (%)	2978/3152 (94.4)	135/146 (92.5)	2841/3006 (94.5)	0.610
Systemic glucocorticoids ever, n/N (%)	638/641 (99.5)	87/90 (96.7)	551/551 (100.0)	0.023
csDMARDs ever, n/N (%)	1786/3152 (56.7)	123/146 (84.2)	1663/3006 (55.3)	0.023
bDMARDs ever, n/N (%)	1836/3152 (58.2)	120/146 (82.2)	1716/3006 (57.1)	0.023

All results are presented as mean and SD and percentages for continuous and categorical variables, respectively.

bDMARDs: biological disease-modifying antirheumatic drugs; B-H Adj. *P*: Benjamini-Hochberg adjusted *P*-value; BMI: body mass index; CRP: C reactive protein; csDMARDs: conventional synthetic disease-modifying antirheumatic drugs; IBD: inflammatory bowel disease; NSAIDs: non-steroidal anti-inflammatory drugs.

We performed a comparative analysis between the groups SpA and IBD and an expanded IBD group including patients diagnosed with IBD-arthritis (group excluded from the main analysis) to identify any clinical differences that the inclusion of the IBD-arthritis diagnosis might reveal in the context of SpA. This additional analysis revealed no major differences in the clinical profiles between the two groups (see Supplementary Table S1, available at *Rheumatology Advances in Practice* online).

### Demographic characteristics within CD and UC subgroups

Among the 146 patients diagnosed with IBD and confirmed with endoscopy, 87 (59.6%) patients were classified into CD and 39 (26.7%) patients into UC; 20 (13.7%) remaining patients with IBD were classified as “no specification for IBD subtype”. Age and BMI were similar across the three groups, with a mean (SD) age of 44.9 (13.5) years for CD, 44.0 (13.0) for UC and 44.7 (12.8) for other IBDs; and BMI between 25 and 27, stating that in average, patients were overweighted according to the WHO classification for BMI [20]. Similarly to the whole cohort, male were more predominant in both groups (63.2% in CD group and 51.3% in UC group). Smoking history was similar across the groups (41.4% CD, 48.7% UC and 50% other IBD) while alcohol consumption varied with higher prevalence by the CD group (43.7% CD, 25.6% UC and 15% other IBD). Regarding geographical distribution, UC was more prevalent in Latin America and there were nearly no patients in Asia with any of the three forms of IBD (see Table 2).

**Table 2.** Socio-demographics and clinical characteristics, disease activity and treatment of patients with SpA stratified by presence of Crohn’s disease or ulcerative colitis

	Crohn’s disease *N* = 87	Ulcerative colitis *N* = 39	**B-H Adj. *P*** ^a^	Other IBDs *N* = 20	**B-H Adj. *P*** ^b^
**Demographics**					
Age, years, mean (SD)	44.9 (13.5)	44.0 (13.0)	0.802	44.7 (12.8)	0.0586
Sex, men, n/N (%)	55/87 (63.2)	20/39 (51.3)	0.553	10/20 (50.0)	0.610
BMI, kg/m^2^, mean (SD)	26.9 (5.3)	25.6 (4.6)	0.553	25.1 (2.7)	0.794
Ever smoker, n/N (%)	36/87 (41.4)	19/39 (48.7)	0.682	10/20 (50.0)	0.795
Ever alcohol, n/N (%)	38/87 (43.7)	10/39 (25.6)	0.327	3/20 (15.0)	0.151
Region, n/N (%)			0.089		0.438
Latin America	2/87 (2.3)	6/39 (15.4)		3/20 (15.0)	
Europe and North America	43/87 (49.4)	20/39 (51.3)		10/20 (50.0)	
Asia	0/87 (0.0)	1/39 (2.6)		1/20 (5.0)	
Middle East and North Africa	42/87 (48.3)	12/39 (30.8)		6/20 (30.0)	
Symptom duration of SpA, years, mean (SD)	16.7 (9.4)	16.4 (10.4)	0.790	19.7 (12.6)	0.675
Diagnosis delay of SpA, years, mean (SD)	7.0 (6.9)	8.8 (8.1)	0.653	11.0 (9.1)	0.618
**Extramusculoskeletal involvement**					
Psoriasis ever, diagnosed by a physician, n/N(%)	9/87 (10.3)	6/39 (15.4)	0.698	6/20 (30.0)	0.532
Uveitis ever, n/N (%)	17/87 (19.5)	11/39 (28.2)	0.591	3/20 (15.0)	0.679
**Musculoskeletal involvement**					
Peripheral arthritis ever, n/N (%)	42/87 (48.3)	18/39 (46.2)	0.890	15/20 (75.0)	0.357
Enthesitis ever, n/N (%)	26/87 (29.9)	14/39 (35.9)	0.710	11/20 (55.0)	0.433
Dactylitis ever, n/N (%)	3/87 (3.4)	1/39 (2.6)	0.873	2/20 (10.0)	0.646
Axial involvement ever according to the rheumatologist, n/N (%)	79/87 (90.8)	37/39 (94.9)	0.678	20/20 (100.0)	0.607
Back pain, n/N (%)	84/87 (96.6)	37/39 (94.9)	0.794	20/20 (100.0)	0.770
Sacroiliitis on X-ray, n/N (%)	64/87 (73.6)	26/39 (66.7)	0.540	7/20 (35.0)	0.089
Sacroiliitis on MRI, n/N (%)	46/60 (76.7)	22/32 (68.8)	0.676	12/14 (85.7)	0.681
**Laboratory assessment**					
HLA-B27 positive, n/N (%)	28/54 (51.9)	13/33 (39.4)	0.570	6/18 (33.3)	0.611
Rheumatoid factor positive, n/N (%)	2/57 (3.5)	2/32 (6.3)	0.742	1/18 (5.6)	0.890
CRP mg/l, mean (SD)	11.1 (33.8)	15.3 (30.1)	0.459	32.0 (74.8)	0.457
**Disease activity, function, pros**					
ASDAS-CRP, mean (SD)	2.4 (1.0)	2.4 (1.1)	0.896	2.9 (1.3)	0.501
BASDAI, mean (SD)	3.7 (2.3)	3.2 (2.1)	0.581	4.8 (2.1)	0.790
PGA, mean (SD)	4.3 (2.7)	3.9 (2.6)	0.706	5.0 (2.6)	0.694
BASFI, mean (SD)	3.3 (2.6)	2.2 (2.1)	0.131	4.5 (2.8)	0.871
ASAS-HI, mean (SD)	7.0 (4.5)	5.7 (4.6)	0.443	8.8 (4.4)	0.926
EQ-5D, mean (SD)	0.7 (0.2)	0.7 (0.2)	0.618	0.5 (0.3)	0.828
Fibromyalgia (according to FiRST score), n/N (%)	20/85 (23.5)	6/37 (16.2)	0.658	5/18 (27.8)	0.741
**Treatment**					
NSAIDs, n/N (%)	79/87 (90.8)	36/39 (92.3)	0.867	20/20 (100.0)	0.656
Systemic glucocorticoids ever, n/N (%)	47/49 (95.9)	23/24 (95.8)	0.990	17/17 (100.0)	0.801
csDMARDs ever, n/N (%)	71/87 (81.6)	35/39 (89.7)	0.578	17/20 (85.0)	0.706
bDMARDs ever, n/N (%)	72/87 (82.8)	33/39 (84.6)	0.871	15/20 (75.0)	0.793

All results are presented as mean and SD and percentages for continuous and categorical variables, respectively.

ASAS-HI: ASAS Health Index; ASDAS: Ankylosing Spondylitis Disease Activity Score; BASDAI: Bath Ankylosing Disease Activity Index; BASFI: Bath Ankylosing Spondylitis Functional Index; bDMARDs: biological disease-modifying antirheumatic drugs; B-H Adj. *P*: Benjamini-Hochberg adjusted *P*-value; BMI: body mass index; CRP: C reactive protein; csDMARDs: conventional synthetic disease-modifying antirheumatic drugs; IBD: inflammatory bowel disease; NSAIDs: non-steroidal anti-inflammatory drugs; PGA: Patient’s Global Assessment.

a Compare with Crohn’s disease and ulcerative colitis.

b Compare with Crohn’s disease and ulcerative colitis and Other IBDs.

### Musculoskeletal manifestations within CD and UC subgroups

In general, SpA patients with CD or UC did not differ in their musculoskeletal phenotyping. Among patients with CD, 75 patients had a diagnosis of axial SpA and 12 of peripheral SpA by their local rheumatologist. Patients with UC presented a similar ratio between SpA classification, with 34 patients with axial SpA and 5 with peripheral SpA (see Fig. 1). The diagnostic delay for SpA was 7.0 (6.9) years for CD and 8.8 (8.1) years for UC group. The most common symptom was chronic back pain (95.4% in CD and 89.7% in UC), defined as back pain longer than 3 months. The presence of peripheral arthritis, enthesitis, and dactylitis was similar among the groups. The distribution of the arthritis was predominantly oligoarthritis with preference on the hands (see Table 2 for details). A third of patients reported history of enthesitis with a clear dominance of the heel enthesitis, present during the study in 3.4% of patients with CD and in 12.8% of patients with UC. CD patients showed a higher tendency to be HLA-B27 positive (51.9% in CD *vs*.s 39.4% in UC), but this did not reach statistical significance. In terms of extra-musculoskeletal involvement other than IBD, UC patients showed a higher frequency of psoriasis and uveitis diagnosis compared with CD patients (15.4% *vs* 10.3% for psoriasis and 28.2% *vs* 19.5% for uveitis; respectively), although this was not statistically significant. Regarding SpA disease activity, such as ASDAS, BASDAI, BASFI and CRP, there were no major differences between patients with CD or UC—see Table 2.

We did a sub-analysis stratifying patients between their predominant SpA form: axial and peripheral and investigated if their phenotyping between those with CD and UC were similar (see Supplementary Table S2, available at *Rheumatology Advances in Practice* online). Patients with axial SpA and CD were more predominantly males than UC (66.7% *vs*.s 52.9%); instead, patients with peripheral SpA were less predominantly male in both IBD forms (41.7% from CD and 40% for UC). Symptom duration, diagnosis delay for SpA and musculoskeletal manifestations were similar in CD and UC patients independently of the form of SpA that they suffered. Psoriasis was more common in CD with axial SpA and, uveitis in patients with UC and peripheral SpA. HLA-B27 positivity was more prevalent in CD patients than in UC patients (58.7% *vs* 41.9% in the axial SpA group; and 12.5% *vs* 0% in the peripheral SpA) – See Supplementary Table S2, available at *Rheumatology Advances in Practice* online.

## Discussion

While the SpA as a whole spectrum has long been in the scientific focus, we investigated a clinical angle that has not been well explored: the similarities and differences in patients with SpA and a parallel diagnosis of CD or UC. Our findings from the ASAS-PerSpA cohort suggest an overall resemblance in demographic characteristics and clinical presentation among SpA patients with either CD or UC.

The comparison of demographics between patients with CD and UC showed no differences, supporting the existing literature that suggests a similar demographic profile among patients diagnosed with either condition [21, 22]. However, we observed a slightly increased prevalence of male patients in the CD group compared with UC. This finding aligns with the mixed results from various studies, showing no definitive consensus on whether sex differences exist between CD and UC [23, 24].

Our study found no differences between CD and UC in relation to musculoskeletal manifestations, with chronic back pain being the predominant symptom in both entities. This similarity in musculoskeletal phenotyping suggests that the presence of CD or UC may not impact the musculoskeletal presentation of SpA. In addition, the distribution of peripheral arthritis, enthesitis, and dactylitis was similar between patients diagnosed with CD or UC, reinforcing the perception of comparable musculoskeletal involvement. Although no prior studies have specifically investigated how IBD might influence SpA’s presentation, our results are in sync with studies that explored the presence of musculoskeletal involvement in patients with IBD. In this area, several studies showed no substantial differences in the articular manifestations based on whether the patients were classified under CD or CU [25–29].

Regarding other extra-musculoskeletal manifestations, our data suggested higher prevalence of both uveitis and psoriasis in the UC group compared with the CD group, although it was not statistically significant. While anterior acute uveitis, an inflammatory eye condition, is recognized as the most common extra-musculoskeletal manifestation in SpA with a prevalence of up to 33% [30], the differential prevalence between CD and UC within the context of SpA claims for deeper exploration. Existing literature presents unconclusive data, with more recent studies describing a higher prevalence in CD over UC [31–34]. These differences might be related to heterogeneity in their methodology and that they are based on single populations, whereas our study is a worldwide cohort.

Interestingly, we observed a trend towards a higher frequency of HLA-B27 positivity among patients with CD compared with UC. In any case, our observation should be interpreted with caution and it needs further analysis to confirm this association. In an extension arm of the German Spondyloarthritis Inception Cohort (GESPIC) the prevalence of HLA-B27 in patients with CD was similar to healthy population [35], but to our knowledge, the most of the studies have explored HLA-B27 status in the conjunction of IBD [28, 36, 37]. Only one study [38] has previously described an HLA-B27 comparison, where 33.3% of patients with CD were HLA-B27 positive compared with 25% of patients with UC; however, the HLA-B27 positivity in those patients was much higher than the general population, bringing some controversy to the literature. HLA-B27 is a genetic marker that has been strongly associated with the development of SpA, meaning a strong genetic component to the disease’s aetiology [39, 40]. Specific interactions between the HLA-B27 gene and other genetic or environmental factors might predispose individuals with CD to develop SpA more than those with UC. For example, the HLA-B27 expression might interact with the gut microbiota associated with CD, leading to aberrant immune responses that could contribute to SpA [41, 42]. If future research confirms our findings and demonstrates a higher frequency of HLA-B27 positivity in patients with CD compared with UC and the development of SpA, it could indicate a distinct genetic or immunological connection between CD, HLA-B27 and SpA. This would not only strengthen our understanding of the pathophysiology of the diseases but also potentially facilitate the diagnosis of the concomitant diseases and the development of more targeted and effective therapeutic strategies.

This study presents several strengths and limitations that should be considered when interpreting the results. A key strength is the international scope of the study, which was conducted in 24 countries, enabling the collection of diverse population and at the same time bringing generalizability of the results to different populations. However, the cross-sectional nature of the study is a limiting factor, leading to interpret the observed associations as correlations rather than causal relationships. The use of endoscopy-based criteria for CD or UC diagnosis, while robust, could potentially overlook patients and it left 20 patients out of the classification due to inconclusive or missing data. Although ASAS-PerSpA study included over 3000 patients, making it a large cohort, the small sample size with patients with concomitant CD and UC may have reduced the statistical power to detect differences between the two IBD entities, and could be an explanation why trends, such as higher prevalence of HLA-B27 among patients with CD did not reach statistical significance.

Our study explored how clinical manifestations presented in patients with SpA and concomitant CD or UC, finding no major phenotypic discrepancies. Although this may not impact the initial diagnosis of SpA, it is essential in the management and treatment of patients with SpA, as each IBD entity requires distinct therapeutic approaches. Further research is needed to optimize patient care and develop more personalized treatment strategies, along with deeper understanding of the shared pathogenetic mechanisms between these conditions. This study contributes to the body of knowledge in rheumatology and gastroenterology, highlighting the crucial role of interdisciplinary approach in managing patients with concurrent SpA and IBD.

## Supplementary Material

rkae064_Supplementary_Data

## Data Availability

The data supporting the results of this study are available from the corresponding author (VRR) upon reasonable request.
