# Supplementary material for: Comparing clinical profiles in spondyloarthritis with Crohn’s disease or ulcerative colitis: insights from the ASAS-PerSpA study
Source: Rheumatol Adv Pract. 2024 May 22;8(2):rkae064. doi: 10.1093/rap/rkae064 (PMC11132819; doi:10.1093/rap/rkae064)
Supplement: rkae064_Supplementary_Data [file rkae064_supplementary_data.docx]

**Supplementary Table S1. Demographic and clinical characteristics including disease activity and treatment of patients with primary diagnosis of SpA and concomitant IBD versus patients with primary diagnosis of IBD- arthritis and concomitant SpA.**

|  | **Patients with SpA and IBD**  **N=146** | **Patients with IBD and Arthritis**  **N=111** | **B-H Adj. P** |
| --- | --- | --- | --- |
| **DEMOGRAPHICS** |  |  |  |
| Age, mean (SD) | 44.6 (13.2) | 46.7 (14.3) | 0.247 |
| Sex (men), n/N (%) | 85/146 (58.2) | 59/ 111(53.2) | 0.350 |
| BMI (kg/m^2^), mean (SD) | 26.3 (4.9) | 24.6 (4.9) | 0.557 |
| Ever smoker, n/N (%) | 65/146 (44.5) | 45/111 (40.5) | 0.694 |
| Ever alcohol, n/N (%) | 51/146 (34.9) | 42/111 (37.8) | 0.529 |
| Symptom duration of SpA (years), mean (SD) | 17.0 (10.1) | 14.8 (10.9) | 0.023 |
| Diagnosis delay of SpA (years), mean (SD) | 8.1 (7.6) | 9.0 (9.1) | 0.023 |
| **EXTRAMUSCULOSKELETAL INVOLVEMENT** |  |  |  |
| Personal history of psoriasis diagnosed by a physician, n/N (%) | 21/146 (14.4) | 4/111 (3.6) | 0.023 |
| Uveitis ever, n/N (%) | 31/146 (21.2) | 18/111 (16.2) | 0.975 |
| **MUSCULOSKELETAL INVOLVEMENT** |  |  |  |
| Peripheral articular disease ever, n/N (%) | 75/146 (51.4) | 77/111 (69.4) | 0.356 |
| Enthesitis ever, n/N (%) | 51/146 (34.9) | 54/111 (48.6) | 0.275 |
| Dactylitis ever, n/N (%) | 6/146 (4.1) | 10/111 (9.0) | 0.337 |
| Axial involvement ever according to the rheumatologist, n/N (%) | 136/146 (93.2) | 76/111 (68.5) | 0.707 |
| Back pain, n/N (%) | 141/146 (96.6) | 86/111 (77.5) | 0.678 |
| Sacroiliitis on X-ray, n/N (%) | 97/146 (66.4) | 50/106(45.0) | 0.804 |
| Sacroiliitis on MRI, n/N (%) | 80/106 (75.5) | 37/67 (55.2) | 0.780 |
| **LABORATORY ASSESSMENT** |  |  |  |
| HLA-B27 positive, n/N (%) | 47/105 (44.8) | 11/57 (19.3) | 0.023 |
| Rheumatoid factor positive, n/N (%) | 5/107 (4.7) | 2/86 (2.3) | 0.464 |
| CRP mg/L, mean (SD) | 15.1 (41.2) | 9.4 (16.4) | 0.578 |
| **DISEASE ACTIVITY, FUNCTION, PROs** |  |  |  |
| ASDAS-CRP, mean (SD) | 2.5 (1.1) | 2.4 (1.0) | 0.796 |
| BASDAI, mean (SD) | 3.7 (2.3) | 3.6 (2.3) | 0.924 |
| PGA, mean (SD) | 4.3 (2.6) | 4.2 (2.7) | 0.981 |
| BASFI, mean (SD) | 3.2 (2.6) | 2.8 (2.7) | 0.511 |
| ASAS-HI, mean (SD) | 6.9 (4.6) | 6.4 (4.3) | 0.454 |
| EQ-5D, mean (SD) | 0.6 (0.2) | 0.76(0.2) | 0.612 |
| Fibromyalgia (according to FiRST score), n/N (%) | 31/140 (22.1) | 15/109 (13.8) | 0.457 |
| **TREATMENT** |  |  |  |
| NSAIDs, n/N (%) | 135/146 (92.5) | 83/111 (74.8) | 0.610 |
| Systemic glucocorticoids ever, n/N (%) | 87/90 (96.7) | 70/82 (85.4) | 0.023 |
| csDMARDs ever, n/N (%) | 123/146 (84.2) | 108/111(97.3) | 0.023 |
| bDMARDs ever, n/N (%) | 120/146 (82.2) | 74/111 (66.7) | 0.023 |

All results are presented as mean and SD and percentages for continuous and categorical variables, respectively.

ASAS-HI, ASAS Health Index; ASDAS, Ankylosing Spondylitis Disease Activity Score; AxSpA, axial spondyloarthritis; BASDAI, Bath Ankylosing Disease Activity Index; BASFI, Bath Ankylosing Spondylitis Functional Index; bDMARDs, biological disease-modifying antirheumatic drugs; B-H Adj. P, Benjamini-Hochberg adjusted p value; BMI, body mass index; CRP, C reactive protein; csDMARDs, conventional synthetic disease-modifying antirheumatic drugs; IBD, inflammatory bowel disease; NSAIDs, Non-steroidal anti-inflammatory drugs; PGA, Patient’s Global Assessment; PsA, psoriatic arthritis; pSpA, peripheral spondyloarthritis.

**Supplementary Table S2. Socio-demographics and clinical characteristics, disease activity and treatment of patients with AxSpA or pSpA with IBD stratified by the presence of Crohn’s disease or ulcerative colitis.**

|  | **AxSpA patients with IBD** | | | | **pSpA patients with IBD** | | | |  |
| --- | --- | --- | --- | --- | --- | --- | --- | --- | --- |
|  | **Total**  **N=127** | **Crohn’s Disease**  **N=75** | **Ulcerative Colitis**  **N=34** | **Other IBDs**  **N=18** | **Total**  **N=19** | **Crohn’s Disease**  **N=12** | **Ulcerative Colitis**  **N=5** | **Other IBDs**  **N=2** | **B-H Adj P*** |
| **DEMOGRAPHICS** |  |  |  |  |  |  |  |  |  |
| Age, years, mean (SD) | 44.1 (12.7) | 44.5 (12.8) | 42.3 (12.8) | 45.5 (12.7) | 48.0 (16.1) | 46.8 (17.9) | 55.3 (9.3) | 36.9 (16.0) | 0.655 |
| Sex (men), n/N (%) | 78/127 (61.4) | 50/75 (66.7) | 18/34 (52.9) | 10/18 (55.6) | 7/19 (36.8) | 5/12 (41.7) | 2/5 (40.0) | 0/2 (0.0) | 0.287 |
| BMI, kg/m^2^, mean (SD) | 26.1 (4.7) | 26.7 (5.3) | 25.2 (4.0) | 25.2 (2.8) | 27.4 (5.7) | 27.6 (5.4) | 28.1 (7.6) | 24.1 (2.5) | 0.677 |
| Ever smoker, n/N (%) | 60/127 (47.2) | 32/75 (42.7) | 18/34 (52.9) | 10/18 (55.6) | 5/19 (26.3) | 4/12 (33.3) | 1/5 (20.0) | 0/2 (0.0) | 0.391 |
| Ever alcohol, n/N (%) | 46/127 (36.2) | 33/75 (44.0) | 10/34 (29.4) | 3/18 (16.7) | 5/19 (26.3) | 5/12 (41.7) | 0/5 (0.0) | 0/2 (0.0) | 0.665 |
| Symptom duration of SpA, years, mean (SD) | 17.3 (10.5) | 16.9 (9.8) | 16.4 (10.8) | 20.8 (12.8) | 14.7 (6.7) | 15.0 (6.7) | 16.0 (7.8) | 9.7 (1.8) | 0.736 |
| Diagnosis delay of SpA, years, mean (SD) | 8.0 (7.8) | 6.9 (7.2) | 8.4 (8.1) | 11.8 (9.2) | 8.3 (6.3) | 7.8 (5.1) | 11.5 (8.9) | 3.0 (2.8) | 0.718 |
| **EXTRAMUSCULOSKELETAL INVOLVEMENT** |  |  |  |  |  |  |  |  |  |
| Psoriasis ever, diagnosed by a physician, n/N (%) | 17/127 (13.4) | 7/75 (9.3) | 6/34 (17.6) | 4/18 (22.2) | 4/19 (21.1) | 2/12 (16.7) | 0/5 (0.0) | 2/2 (100.0) | 0.767 |
| Uveitis ever, n/N (%) | 30/127 (23.6) | 17/75 (22.7) | 10/34 (29.4) | 3/18 (16.7) | 1/19 (5.3) | 0/12 (0.0) | 1/5 (20.0) | 0/2 (0.0) | 0.356 |
| **MUSCULOSKELETAL INVOLVEMENT** |  |  |  |  |  |  |  |  |  |
| Peripheral arthritis ever, n/N (%) | 59/127 (46.5) | 33/75 (44.0) | 13/34 (38.2) | 13/18 (72.2) | 16/19 (84.2) | 9/12 (75.0) | 5/5 (100.0) | 2/2 (100.0) | 0.024 |
| Enthesitis ever, n/N (%) | 37/127 (29.1) | 18/75 (24.0) | 10/34 (29.4) | 9/18 (50.0) | 14/19 (73.7) | 8/12 (66.7) | 4/5 (80.0) | 2/2 (100.0) | 0.023 |
| Dactylitis ever, n/N (%) | 4/127 (3.1) | 2/75 (2.7) | 1/34 (2.9) | 1/18 (5.6) | 2/19 (10.5) | 1/12 (8.3) | 0/5 (0.0) | 1/2 (50.0) | 0.456 |
| Axial involvement ever according to the rheumatologist, n/N (%) | 124/127 (97.6) | 72/75 (96.0) | 34/34 (100.0) | 18/18 (100.0) | 12/19 (63.2) | 7/12 (58.3) | 3/5 (60.0) | 2/2 (100.0) | 0.023 |
| Back pain, n/N (%) | 126/127 (99.2) | 74/75 (98.7) | 34/34 (100.0) | 18/18 (100.0) | 15/19 (78.9) | 10/12 (83.3) | 3/5 (60.0) | 2/2 (100.0) | 0.023 |
| Sacroiliitis on X-ray, n/N (%) | 89/127 (70.1) | 58/75 (77.3) | 24/34 (70.6) | 7/18 (38.9) | 9/19 (47.3) | 6/12 (50.0) | 2/5 (40.0) | 1/2 (50.0) | 0.061 |
| Sacroiliitis on MRI, n/N (%) | 76/92 (82.6) | 44/51 (86.3) | 21/28 (75.0) | 11/13 (84.6) | 4/14 (28.6) | 2/9 (22.2) | 1/4 (25.0) | 1/1 (100.0) | 0.023 |
| **LABORATORY ASSESSMENT** |  |  |  |  |  |  |  |  |  |
| HLA-B27 positive, n/N (%) | 46/93 (49.5) | 27/46 (58.7) | 13/31 (41.9) | 6/16 (37.5) | 1/12 (8.3) | 1/8 (12.5) | 0/2 (0.0) | 0/2 (0.0) | 0.068 |
| CRP mg/L, mean (SD) | 14.4 (41.7) | 11.5 (36.0) | 9.7 (17.3) | 35.5 (78.3) | 19.5 (38.4) | 8.4 (14.9) | 53.8 (63.2) | 0.5 (0.7) | 0.982 |
| **DISEASE ACTIVITY, FUNCTION, PROs** |  |  |  |  |  |  |  |  |  |
| ASDAS-CRP, mean (SD) | 2.4 (1.1) | 2.4 (1.0) | 2.2 (1.0) | 3.1 (1.3) | 2.7 (1.2) | 2.5 (1.1) | 3.6 (1.3) | 1.6 (1.3) | 0.588 |
| BASDAI, mean (SD) | 3.6 (2.2) | 3.6 (2.2) | 2.9 (2.1) | 5.1 (2.0) | 4.1 (2.4) | 4.0 (2.6) | 4.9 (1.8) | 2.7 (3.3) | 0.618 |
| PGA, mean (SD) | 4.1 (2.6) | 4.1 (2.7) | 3.5 (2.5) | 5.2 (2.4) | 5.4 (2.5) | 5.3 (2.4) | 6.4 (2.1) | 3.5 (4.9) | 0.232 |
| BASFI, mean (SD) | 3.1 (2.6) | 3.3 (2.6) | 2.0 (2.0) | 4.7 (2.7) | 3.5 (2.3) | 3.7 (2.3) | 3.6 (2.4) | 2.4 (3.3) | 0.653 |
| ASAS-HI, mean (SD) | 6.6 (4.6) | 6.8 (4.6) | 5.0 (4.3) | 8.9 (3.8) | 8.8 (4.6) | 8.4 (3.8) | 9.9 (4.6) | 8.0 (11.3) | 0.350 |
| EQ-5D, mean (SD) | 0.6 (0.2) | 0.7 (0.2) | 0.7 (0.2) | 0.5 (0.3) | 0.6 (0.2) | 0.6 (0.2) | 0.6 (0.1) | 0.6 (0.6) | 0.559 |
| **TREATMENT** |  |  |  |  |  |  |  |  |  |
| NSAIDs, n/N (%) | 117/127 (92.1) | 68/75 (90.7) | 31/34 (91.2) | 18/18 (100.0) | 18/19 (94.7) | 11/12 (91.7) | 5/5 (100.0) | 2/2 (100.0) | 0.798 |
| Systemic glucocorticoids ever, n/N (%) | 72/74 (97.3) | 38/40 (95.0) | 19/19 (100.0) | 15/15 (100.0) | 15/16 (93.8) | 9/9 (100.0) | 4/5 (80.0) | 2/2 (100.0) | 0.698 |
| csDMARDs ever, n/N (%) | 105/127 (82.7) | 60/75 (80.0) | 30/34 (88.2) | 15/18 (83.3) | 18/19 (94.7) | 11/12 (91.7) | 5/5 (100.0) | 2/2 (100.0) | 0.532 |
| bDMARDs ever, n/N (%) | 104/127 (81.9) | 61/75 (81.3) | 30/34 (88.2) | 13/18 (72.2) | 16/19 (84.2) | 11/12 (91.7) | 3/5 (60.0) | 2/2 (100.0) | 0.876 |

All results are presented as mean and SD and percentages for continuous and categorical variables, respectively.

*Compare with total patient groups

ASAS-HI, ASAS Health Index; ASDAS, Ankylosing Spondylitis Disease Activity Score; AxSpA, axial spondyloarthritis; BASDAI, Bath Ankylosing Disease Activity Index; BASFI, Bath Ankylosing Spondylitis Functional Index; bDMARDs, biological disease-modifying antirheumatic drugs; B-H Adj. P, Benjamini-Hochberg adjusted p value; BMI, body mass index; CRP, C reactive protein; csDMARDs, conventional synthetic disease-modifying antirheumatic drugs; IBD, inflammatory bowel disease; NSAIDs, Non-steroidal anti-inflammatory drugs; PGA, Patient’s Global Assessment; PsA, psoriatic arthritis; pSpA, peripheral spondyloarthritis.
